# A meta-epidemiological study of subgroup analyses in cochrane systematic reviews of atrial fibrillation

**DOI:** 10.1186/s13643-019-1152-z

**Published:** 2019-10-25

**Authors:** Miney Paquette, Ahlam Mohammed Alotaibi, Robby Nieuwlaat, Nancy Santesso, Lawrence Mbuagbaw

**Affiliations:** 1 Department of Health Research Methods, Evidence, and Impact, McMaster University, Hamilton, Ontario L8S 4K1 Canada; 2 Medical Department, Boehringer Ingelheim Ltd., Burlington, Ontario Canada; 3 Pediatric Endocrinology Department, King Abdullah bin Abdulaziz University hospital, Princess Noura University, Riyadh, Saudi Arabia; 4 Biostatistics Unit, Father Sean O’Sullivan Research Centre, St Joseph’s Healthcare Hamilton, Hamilton, Ontario Canada; 5 Centre for the Development of Best Practices in Health, Yaoundé, Cameroon

**Keywords:** Meta-epidemiological review, Atrial fibrillation, Subgroup analyses

## Abstract

**Background:**

Information on subgroup assessments in systematic reviews (SR) of atrial fibrillation (AF) is limited. This review aims to describe subgroup analyses in AF SRs to inform the design of SRs and randomized trials as well as clinical practice.

**Methods:**

We conducted a cross sectional meta-epidemiological study of Cochrane AF reviews by searching AF (including variants) in the title, abstract, or keyword field without date or language restrictions (Issue 9; September 2018). Two reviewers independently extracted study characteristics to summarize frequency of subgroups pre-specified and conducted and report credibility of subgroup effects claimed.

**Results:**

Of 39 Cochrane reviews identified, 17 met inclusion criteria (including 168 reports of 127 randomized trials) and the majority (16; 94.1%) conducted meta-analysis of outcomes. Most (13; 76.5%) planned pre-specified subgroup analyses; 7 of which (41.2%) conducted subgroups. In these 7 reviews, 56 subgroups were planned, 17 (30.4%) conducted and 6 (10.7%) yielded subgroup effects. Variables such as co-morbid disease, stroke risk factors, prior stroke/transient ischemic attack, age, race, and sex represented 44% (24 subgroups) of all planned subgroups (8 conducted; 14.3%); however, information on covariate selection was lacking. Overall, more subgroups were planned than conducted (mean difference (95% CI) 2.3 (1.2–3.5, *p* < 0.001)). Of all subgroups conducted, anticoagulant characteristics comprised a third of all subgroup effects (*n* = 5, 35.7%).

The credibility of subgroups identified (*n* = 14) was assessed and less than half (43%) represented one of a small number of pre-specified hypothesis and rarely were effects seen within studies (7%). Of 5 reviews that reported subgroup effects, only 3 discussed subgroup effects as part of the overall conclusions; none discussed credibility of subgroup effects.

**Conclusions:**

This meta-epidemiological review of a subset of Cochrane AF reviews suggests that planning and reporting of subgroup analyses in AF reviews can be improved to better inform clinical management. Most pre-specified subgroup analyses were not performed, important variables (such as stroke, bleeding risk, and other comorbidities) were rarely examined and credibility of subgroup effects claimed was low. Future reviews should aim to identify important subgroups in their protocols and use recommended approaches to test subgroup effects in order to better support clinical decision-making.

## Background

Systematic reviews (SR) comprised of studies evaluating a central research question are considered to be the pinnacle of the medical evidence hierarchy [1]. To evaluate effects of interventions, combining studies and synthesizing results can provide greater confidence of treatment effects to draw conclusions than individual studies could provide in isolation [2]. Subgroup analyses may be conducted in SRs by dividing data across studies into groups based on participant or study characteristics in order to compare them or to partition out sources of heterogeneity from an overall effect, providing evidence to better guide clinical decision making compared to broad summaries of effects across diverse types of studies and participants [2, 3]. The latter may not confidently inform about optimal treatments for individual or specific groups of patients [4]. Although subgroup analyses may be helpful to understand potential differences in patient or study characteristics, such investigations are uncommon because sufficient information is typically not available in published reports. Furthermore, interpretation should be made appreciating the risk for increasing probability of type 2 error concurrent with the number of subgroup analyses conducted [2].

Clinical heterogeneity is a term broadly used to include patient variability in clinical attributes as well as treatment variability that can include factors such as timing, formulation, doses, and duration, as well as variability in the settings in which treatments are delivered and measurement of outcomes [4]. Variability in study designs or quality of the studies is referred to as methodological heterogeneity, and together these sources of heterogeneity contribute to statistical heterogeneity, characterized by different magnitudes of treatment effect observed between studies in meta-analysis [4]. Consistency in effects support overall quality of evidence and tools for evaluating consistency include measures of heterogeneity such as *I*-squared (*I*^2^) [5], extent of overlap in confidence intervals and similar point estimates of effect [6]. Meta-regression is another method for assessing the relationship between one or more study level covariates and the effect size in studies which accommodates continuous, as well as categorical covariates and allows consideration of multiple covariates in the same model when there are adequate numbers of studies [7]. Several papers have been published, which emphasize the importance of evaluating and incorporating identified heterogeneity in the final interpretation of results [3, 4, 8, 9].

### Planning and interpretation of subgroup analyses

Analyses of subgroups can help elucidate key sources of clinical heterogeneity; however, sufficient guidance on which important subgroups should be examined is lacking for specific disease areas. Generally, the most important subgroups to examine should be pre-specified based on presumed or known relationships to outcomes but standardized approaches to determining which subgroups to investigate do not exist. Broad recommendations for investigating clinical heterogeneity have been proposed such as ensuring that evaluations are pre-specified, with clear rationale [4]. Furthermore, as there is risk for spurious subgroup effects, guidance has been published to help assess credibility of subgroup effects by evaluating 5 key criteria including the establishment of a limited number of important subgroups which are pre-specified according to some biological basis [10].

Previous meta-epidemiological reviews have identified other limitations of SRs such as suboptimal application of statistical testing principles with low proportions of reviews reporting appropriate interaction effect testing [11, 12] and minimal discussion of implications for possible confounding within subgroup analyses [11–13].

### Subgroup analyses in atrial fibrillation

Systematic reviews of AF have not been explored to describe the extent to which subgroup analyses are pre-specified and conducted, nor have important subgroups across AF reviews been reported. AF patients have diverse co-morbidities such as coronary artery disease, diabetes, heart failure, and hypertension, as well as variability in frequency and patterning of AF episodes and symptom burden [14]. In prospective studies, it has been demonstrated that advanced age, female sex and co-morbid diseases such as diabetes, heart failure, prior stroke, or transient ischemic attack are independent risk factors for stroke [15, 16]. Broader evaluation of additional sources of heterogeneity that may contribute to overall treatment differences could provide important insights to guide optimal management of this increasingly prevalent arrhythmia which is well recognized as an important independent risk factor for ischemic stroke [17].

Factors identified that increase stroke risk (for example, advanced age and co-morbid diseases such as impaired left ventricular systolic function) [18, 19] may be of particular interest in systematic reviews of AF. Other clinical factors such as bleeding risk may also be subgroups of interest due to their link to important outcomes [20]. How often these, or other important subgroup analyses, are planned and conducted in AF SRs and which subgroup effects are associated with important outcomes has not been systematically established. Furthermore, whether subgroup effects are included in the final conclusions has not been reported. Finally, the quality of reviews and whether quality is associated with subgroup analyses planned or conducted has not been examined.

As little is known about which subgroups are most important to explore in AF SRs or if there are any potential inherent limitations to subgroup analyses reported in SRs in AF, a meta-epidemiological review was undertaken. The purpose of this review is to describe subgroup analyses in AF SRs in order to inform the design of systematic reviews and randomized trials as well as clinical practice.

The objectives of this methodological review are to describe subgroup analyses including:

- How often and which subgroup analyses are pre-specified;
- Report subgroup analyses conducted (pre-specified as well as conducted post-hoc);
- Summarize the most frequently identified subgroup effects;
- Assess whether subgroup effects are included in conclusions; and
- Assess credibility of subgroup effects identified [10].

In addition, the quality of reviews using AMSTAR-2 (A MeaSurement Tool to Assess systematic Reviews) criteria [21] will be assessed to determine if subgroups planned or conducted differ with respect to the quality of reviews.

## Methods

We conducted a cross-sectional, meta-epidemiological review from the Cochrane library (Issue 9; September 2018) extracting information from the eligible systematic reviews. We focused this review only on Cochrane reviews because of their methodological and reporting consistency. There were no sample size calculations as we included all the eligible studies in our sampling frame. Reporting of this review was conducted in general consideration of published recommendations and guidelines for reporting meta-epidemiological methodology research [22] (see Additional file 1 for additional details). A protocol was not registered for this review.

Subgroup analyses were considered pre-specified if covariates were included in a registered protocol for the review or if it was explicitly stated in the review that it was pre-specified prior to data collection. Post hoc analyses were defined as analyses which were not included as planned subgroups in protocols and for which there was no mention of pre-specification in the SR report or if they were explicitly identified as post hoc evaluations.

### Search

We searched the Cochrane Database of Systematic Reviews for reviews current to 5 Sept. 2018 (https://www.cochranelibrary.com/advanced-search) using “atrial fibrillation” in the title, abstract, or keyword fields without date, language, study type, or other filters [“atrial fibrillation:ti,ab,kw”; including word variants for atrial fibrillation]. The studies were independently screened by two authors. The selection of articles was conducted in duplicate with any discrepancies of included reviews to be reviewed and assessed by a third author. Separate searches of the Cochrane Library of registered protocols using the term “atrial fibrillation” (including variants and without any “date”, “status”, “language”, “type” or “topic” filters) and the International Prospective Register of Systematic Reviews at https://www.crd.york.ac.uk/PROSPERO/ (PROSPERO) [23] were conducted to retrieve protocols which were available as of the search date on 4 Nov. 2018. Authors were not contacted for confirmation of data or missing information.

### Eligibility criteria and selection

Cochrane systematic reviews in AF patients were included irrespective of study design of included studies, or whether the category of review was interventional, prognostic, or diagnostic in nature. AF reviews which did not clearly identify AF patients (e.g., mixed indications for antithrombotic treatment without clear identification of the number of AF patients comprised in the group), or those that evaluated AF as an outcome (e.g., post-operative AF as an outcome) were excluded. As AF assessed postoperatively can represent a transient outcome with patients often returning to sinus rhythm, these SRs were determined to be outside of the scope of this review. Furthermore, as AF was our primary indication and patient group of interest, SRs that did not explicitly target or identify AF sub populations were excluded.

Cochrane reviews can be withdrawn when the question is no longer relevant or if the information is included in another review. As one of the main objectives was to evaluate methodology, we included withdrawn reviews. Where updates to reviews were conducted, the most current version of the review was included. The search results were independently screened by two researchers who were also trained to independently extract the data. The first researcher was trained by a senior researcher and the second researcher was trained by the first. Disagreement and discrepancies in results were discussed and resolved.

### Data collection and analysis

We constructed data collection forms prior to performing the search, extracting key data from the reviews including the author information, year of publication, category of review (e.g. therapeutic/interventional, prevention), study design, primary objectives, number and types of studies included, total number of patients, indications, meta-analyses conducted or not, primary and secondary outcomes, deviations from protocols, number and type of subgroup analyses pre-specified and conducted, reasons for not conducting subgroup analyses, post hoc analyses conducted, and total number of subgroup effects identified (pre-specified and post hoc). Two authors piloted the data collection forms in 5 SRs and made minor revisions to facilitate extraction which was performed in duplicate by the same authors.

Data were summarized as frequency and percentage for categorical variables and means with standard deviations for continuous items. Where data were not normally distributed, median and interquartile ranges (IQR) were presented. Comparisons were quantified using mean differences with 95% confidence interval (CI), for number of pre-specified versus conducted analyses (with paired *t* test used to assess significance). There were no further transformations of the data or imputation of any missing parameters. Data were summarized and analyzed in Microsoft Excel (2016).

The most important subgroups in each SR (*pre-specified or post hoc*) were identified by evaluating the magnitude of subgroup effects using odds ratios and 95% CI. The most frequent subgroup effects identified across reviews were categorized and plotted for all SRs included.

Methodological quality of reviews was assessed using *A M*ea*S*urement *T*ool to *A*ssess Systematic *R*eviews (AMSTAR-2) [21] by 2 independent reviewers. In accordance with AMSTAR-2, we rated the overall quality assessment of the review as high if there were no critical weaknesses in the review, moderate if there was more than one *non*-critical weakness but no critical flaw, low if there was one critical weakness (with or without *non*-critical weakness) or critically low if there was more than one critical flaw with or without non-critical weakness [21].

Reviews assessed as high quality were compared to those of moderate, low, or critically low quality in number of pre-specified subgroup analyses planned and conducted, overall number of subgroup analyses conducted and number of subgroup effects identified. Two-sample, unequal variance *t* tests were conducted to assess if there were any statistically significant differences between high- and moderate-/low-/critically low- quality reviews.

For subgroup effects identified, the credibility of each effect was assessed by one reviewer using the criteria outlined by Sun et al. including whether results could have been due to chance, consistency of subgroups across studies, whether a limited number of important subgroups according to some biological basis were pre-specified, and whether evidence came from within or between study comparisons [10].

## Results

### Systematic review characteristics

**Fig. 1:**
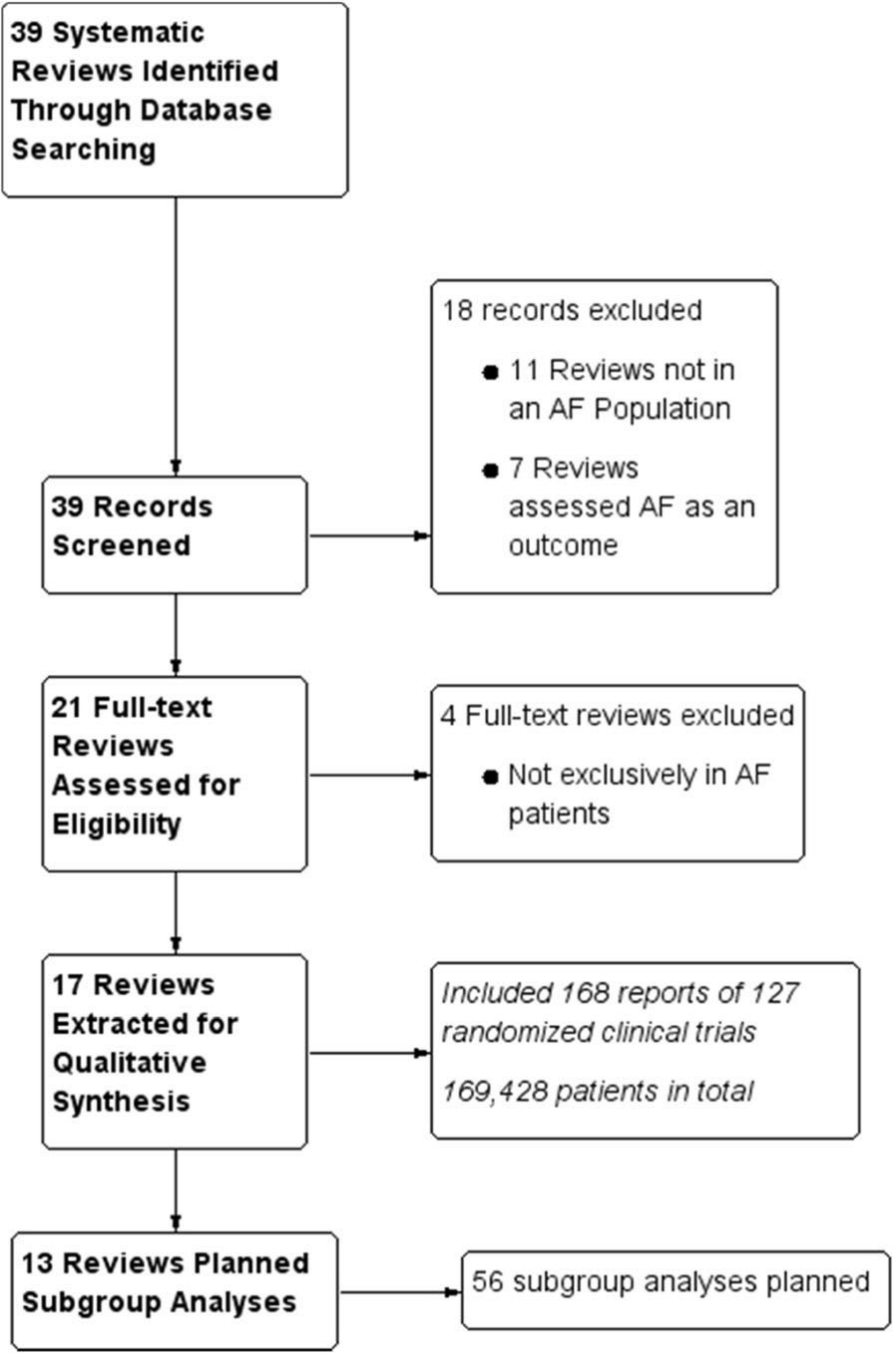
Systematic reviews

We identified a total of 39 systematic reviews and 18 records were excluded following title and abstract review (11 were not in an AF population and 7 assessed AF as an outcome). An additional 4 SRs were excluded based on full-text review as they were not exclusively in an AF population and AF patients could not be clearly differentiated from the total patient population (Fig. 1).

A total of 17 SRs [24–40] which were published between 2004 and 2018 met final eligibility for qualitative and quantitative assessment (Fig. 1). Half of the SRs investigated oral anticoagulants or antiplatelets for stroke prevention (*n* = 9, 52.9%) [24–29, 31, 35, 38]; 4 evaluated surgery, cardioversion, or ablation (23.5%) [33, 37, 40, 41]; 2 evaluated behavioral interventions (11.8%) [30, 36]; 1 (5.9%) was an AF detection review [39]; and 1 (5.9%) evaluated antiarrhythmic medications [34]. The median number of primary outcomes was 2 (IQR 2) and secondary outcomes was 5 (IQR 5). Detailed SR characteristics are presented in the Appendix. One of the SRs was subsequently noted as withdrawn but the report was published and results were available; therefore, information was extracted and included in the quantitative and qualitative assessment [40].

### Subgroup analyses

#### Pre-specified subgroup analyses

All but one [39] of the reviews (*n* = 16; 94.1%) conducted meta-analysis of outcomes. The majority of reviews planned to explore heterogeneity with pre-specified subgroup analyses in 13 of the 17 reviews (76.5%; Table 1) [26, 29–40]. In subsequent evaluation of the protocols and reviews, details were not provided on how pre-specified covariates were selected (or if they comprised a subset of a larger group of pre-specified covariates initially under consideration) and potential for confounding and how it would be managed was not addressed.

**Table 1:** Systematic review characteristics

	Total reviews (*N* = 17)
Reviews with meta-analysis*: *n* (%)	16 (94.1%)
Pre-specified subgroups	
Reviews with pre-specified subgroups^a^	13 (76.5%)
Planned covariates for subgroup analysis: median (min, max)	3 (0, 6)
Reviews with subgroup analyses conducted: *n* (%)^b^	7 (41.2%)
Covariates used in subgroup analyses: median (min, max)	2 (1, 6)
Reviews with subgroup effects**: *n* (%)^c^	3 (17.6%)
Covariates in subgroup analyses: median (min, max)	2 (1, 3)
Post hoc subgroup analyses	
Reviews with post hoc subgroup analyses^d^	5 (29.4%)
Reviews with post hoc subgroup effects: *n* (%) ^e^	4 (23.5%)
Covariates used in subgroup analyses: median (min, max)	1 (1, 5)
Any subgroup analyses performed, pre-specified, or Post Hoc	
Reviews with any subgroup analyses^f^	9 (52.9%)
Covariates used in subgroup analyses: median (min, max)	2 (1, 11)
Number with subgroup effects: *n* (%)	5 (29.4%)
No subgroup analyses performed	
Reviews with no subgroup analyses performed^g^	8 (47.1%)
Reasons for not conducting subgroup analyses: *n* (%)	
Insufficient data/studies	4 (23.5%)
Not planned	4 (23.5%)

*One review [39] did not conduct meta-analysis due to insufficient studies

a[26, 29–40]

b[29, 30, 33, 35, 36, 38, 39]

c[35, 38, 39]

d[24, 30, 31, 35, 38]

e[30, 31, 35, 38]

f[24, 29–31, 33, 35, 36, 38, 39]

g[25–28, 32, 34, 37, 40]

**Sub-group effects were characterized by *p* ≤ 0.05 for interaction terms, using Chi^2^ test for subgroup differences. This does not include 3 reviews which presented effects within a subgroup but did not test for interaction effects.

In these 13 reviews, there were 56 subgroup analyses planned with the most common subgroups being co-morbid disease (chronic kidney disease, diabetes, heart failure/impaired left ventricular dysfunction, 12.5%); anticoagulant type, dose, or quality (12.5%); type of AF (paroxysmal and persistent, 10.7%); age (10.7%); stroke risk scores (8.9%); and sex (8.9%). Co-morbid disease, stroke risk factors, prior stroke/transient ischemic attack (TIA), age, and sex are important subgroups based on their established relationships to outcomes [18–20], yet they represented only 42.8% (24 subgroups) of all planned subgroup analyses of which 14.3% were conducted (8 subgroups).

**Fig. 2:**
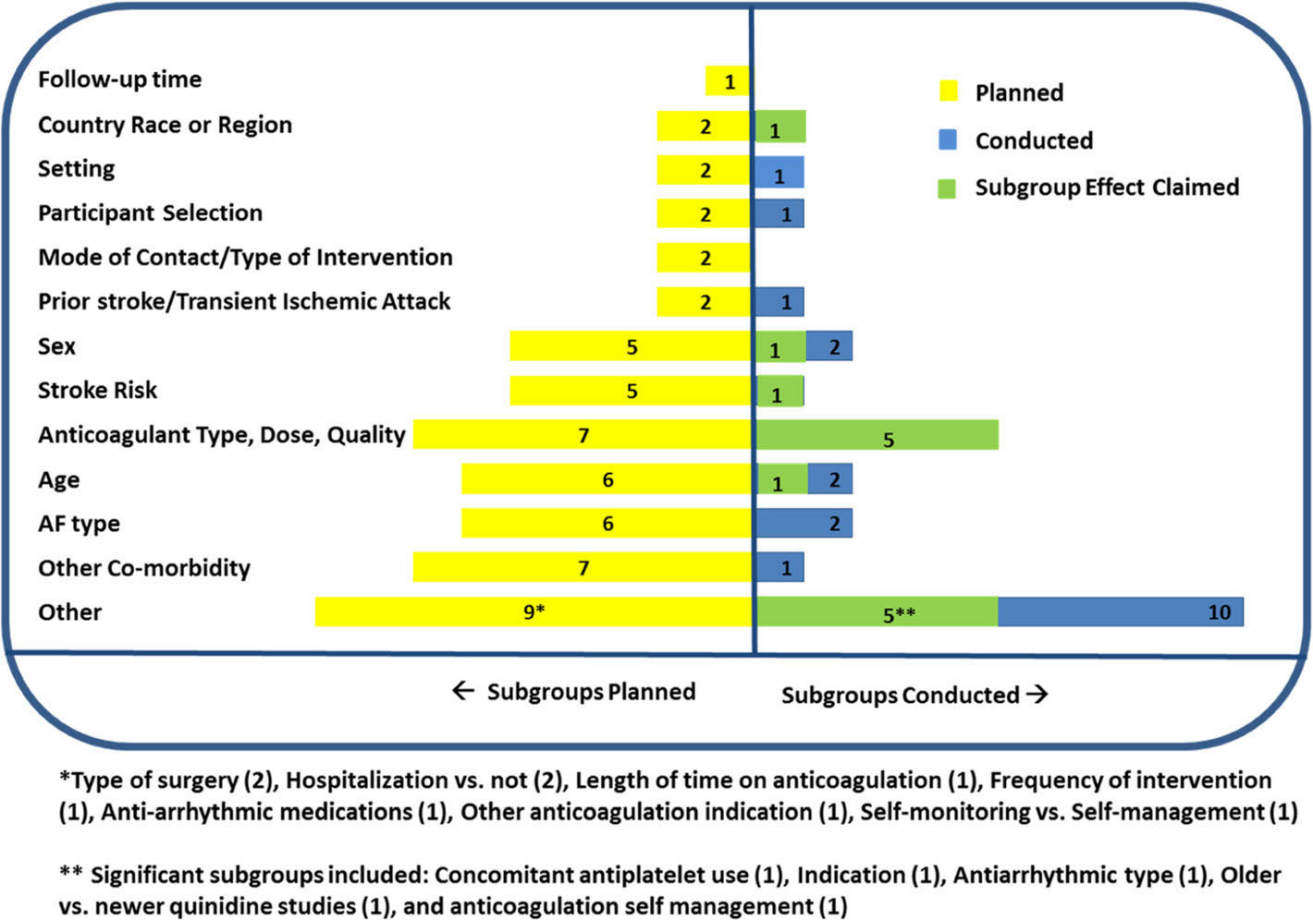
Number of subgroup analyses planned and conducted

Of the 13 reviews that planned to conduct a total of 56 pre-specified analyses, 7 reviews [3, 29, 30, 35, 36, 38, 39] conducted a total of 17 (30.4%) subgroup analyses, of which 3 reviews (17.6%) [35, 38, 39] yielded a total of 6 subgroup effects (Table 2). The mean difference (95% CI) between the number of planned and conducted subgroup analyses per SR was 2.3 (1.2–3.5, *p* < 0.001), and the reasons for not conducting subgroup analyses (*n* = 39) were insufficient studies or lack of data (92.7%) or were not further explained (7.3%). Many of the important planned subgroup analyses such as stroke risk, sex, age, or co-morbidities, such as heart failure or diabetes, were not conducted (Fig. 2).

**Table 2:** Subgroup characteristics

Total subgroup analyses planned or conducted (*n* = 66)	
Pre-specified subgroup analyses	*56*
Pre-specified subgroup analyses conducted (% of planned)	*17 (30.4%)*
Subgroup effects reported (% of pre-specified conducted)	*6 (35.3%)*
Stroke risk (*n*)^a^	1 (1.8%)
Age (*n*)^b^	1 (1.8%)
Sex (*n*)^c^	1 (1.8%)
Country/race (*n*)^d^	1 (1.8%)
Indication (*n*)^e^	1 (1.8%)
Anticoagulation self-management (*n*)^f^	1 (1.8%)
Post hoc subgroup analyses conducted	*10*
Subgroup effects reported (% of post hoc conducted)	*8 (80%)*
Anticoagulation: dose, route, type, or quality (*n*)^g^	5 (50.0%)
Type of antiarrhythmic (*n*)^h^	1 (10.0%)
Older and newer quinidine studies (*n*)^i^	1 (10.0%)
Concomitant antiplatelet use (*n*)^j^	1 (10.0%)
Total pre-specified or post hoc subgroup analyses conducted	*27*
Subgroup effects reported (% of all conducted)	*14 (51.9%)*

a[35]

b[35]

c[39]

d[35]

e[38]

f[38]

g[31, 35, 38]

h[30]

i[35]

For reviews that did not conduct subgroup analyses, the median (IQR) of included studies and patients was 3.0 (4.5) and 1149.0 (1894.8) respectively, compared to a median of 11.0 (17.0) studies and 9137.0 (18,992.0) patients in reviews that conducted subgroup analyses (p = 0.05, and p = 0.01 respectively for comparisons by Mann-Whitney U test).

#### Post hoc subgroup analyses

A total of 10 post hoc subgroup analyses were conducted in 5 reviews (29.4%) [24, 30, 31, 35, 38]; the majority of these comparisons (80%) showed subgroup effects. These included anticoagulant characteristics (including type, dose, quality, or route, *n* = 5), antiarrhythmic drug class (*n* = 1), older and newer quinidine studies (*n* = 1), and concomitant antiplatelet use (*n* = 1).

### Subgroup effects identified

#### All conducted subgroup analyses

In 9 reviews [24, 29–31, 33, 35, 36, 38, 39], a total of 27 pre-specified or post hoc analyses were conducted, of which half yielded subgroup effects (*n* = 14; 51.9%). Sub-group effects were characterized by *p* ≤ 0.05 for the interaction term using Chi^2^ test for subgroup differences. There were 3 reviews which presented effects within a subgroup but did not test for interaction effects [29, 30, 33]. The subgroup effects are further described in Table 2. The most frequent subgroup effect was related to characteristics of anticoagulation (including dose, quality, route, or type), comprising 5 subgroups representing 35.7% of all subgroup effects. The subgroup analyses planned and conducted and subgroup effects reported are shown in Fig. 2.

Studies with subgroup effects related to anticoagulation examined outcomes of stroke and bleeding. For example, in a review conducted by Bruins et al. [35] exploring Factor Xa inhibitors, there were differences in effect size estimates for patient important outcomes such as major bleeding events depending on the type of Factor Xa inhibitor given. The odds ratios for major bleeding compared to vitamin K antagonists (VKA) for some anticoagulants such as apixaban (odds ratio (OR), 95% CI 0.69 (0.60, 0.80)) and betrixaban (OR 0.19, 95% CI 0.05, 0.82) demonstrated a significant risk reduction compared to VKA. In contrast, idraparinux showed increased risk (OR 2.62, 95% CI 1.70, 4.03) compared to VKA. The subgroup interaction effect was significant (Chi^2^ = 63.01, *p* < 0.01) with *I*^2^ = 90% indicating a high degree of heterogeneity attributable to type of Factor Xa inhibitor. In this review, dose and quality of anticoagulation (based on median time-in-therapeutic range ≤ 60% (“low/bad”) versus > 60% (“high/good”) quality treatment) as well as the route of administration showed evidence of heterogeneity in effect. Furthermore, the effect size for the outcomes of stroke and systemic embolic events were highly variable as a function of dose even within the same compound class.

The subgroup of older patients (≥ 75 years) showed greater risk reduction than younger patients (< 75 years) for stroke/systemic embolic events with Factor Xa inhibitors compared to VKA [35] (OR 0.76, 95% CI 0.66, 0.88 and OR 0.96, 95% CI 0.84, 1.09 for older and younger patients, respectively) with subgroup interaction effects (Chi^2^ = 5.07, *p* = 0.02), and high heterogeneity (*I*^2^ = 80%). Other subgroup effects identified included race (Asian, White, Black, and other) [35], as well as CHADS_2_ stroke risk score [42] (comprised of congestive heart failure, hypertension, age ≥ 75 years, diabetes mellitus, and prior stroke/TIA, or thromboembolism) [35], sex [39], and patient self-management of anticoagulation [38]

### Quality of reviews

#### AMSTAR-2 quality of reviews

The methodological quality of reviews was assessed as high in 8 reviews (47.1%) [29, 31, 32, 34, 36–39] and low in 9 reviews (52.9%) [24–28, 30, 33, 35, 40]. The main reason for SRs being assessed as low quality was due to incomplete assessment of risk of bias domains, and/or failure to discuss risk of bias in conclusions where bias was identified. Most SRs assessed as being of low quality only assessed the allocation concealment aspect of risk of bias and did not assess other domains such as random sequence generation, blinding of participants or personnel, blinding of outcome assessment, incomplete outcome data, or other types of bias [43]. Many SRs also did not meet criteria for a comprehensive search strategy as they failed to look at other sources such as gray literature or consultation with experts. The risk of bias assessment for the included reviews is presented in Additional file 2: AMSTAR-2 Risk of Bias for Included Systematic Reviews.

When comparing the quality of SRs between more recent reviews (2016 to Sept 2018) and earlier reviews (prior to 2016), there was a higher proportion of recent reviews that were considered of high quality (75.0%), compared to earlier reviews (22.2%) (Chi^2^ = 4.74, df = 1, *p* = 0.03). In addition, there was a higher average number of planned subgroup analyses in the high quality reviews compared to low quality reviews (mean (SD), 4.75 (1.58) and 2.00 (2.45), respectively, *p* = 0.01), but there were no significant differences in the overall number of subgroup analyses conducted (pre-specified or post hoc) or number of subgroups identified (Table 3).

**Table 3:** Subgroup differences and quality of reviews

Quality	Subgroup analyses					
	Planned			Post hoc		Planned or post hoc
	Planned	Conducted	Subgroup effects	Conducted	Subgroup effects	Subgroup effects
Low: mean (SD)	2.00 (2.45)	1.11 (2.09)	1.50 (2.12)	0.33 (0.50)	1.75 (2.36)	3.25 (4.48)
High: mean (SD)	4.75 (1.58)	0.88 (0.99)	0.75 (0.96)	0.25 (0.46)	0.29 (0.49)	0.63 (1.06)
*p* value (T-test)	0.02	1.00	0.71	0.62	0.30	0.57

*SD* standard deviation

#### Credibility of subgroup analyses

**Fig. 3:**
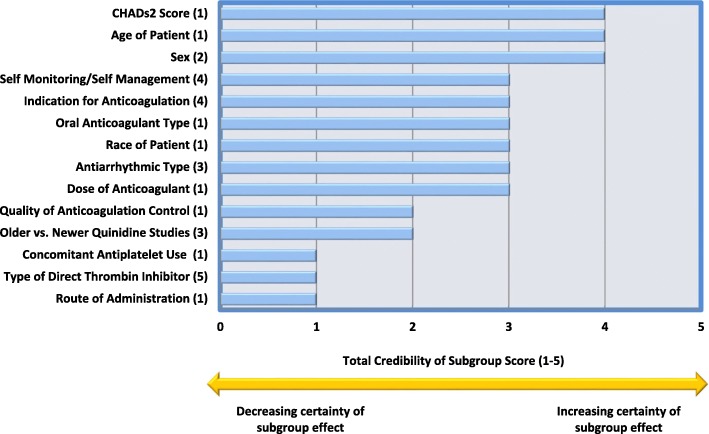
Credibility of individual subgroup effects observed in each review. 1, Bruins et al. [35]; 2, Moran et al. [39]; 3, Lafuente-Lafuente et al. [30]; 4, Heneghan et al. [38]; 5, Salazar et al [31]. Credibility is assessed using the criteria established by Sun et. al. [10]

**Fig. 4:**
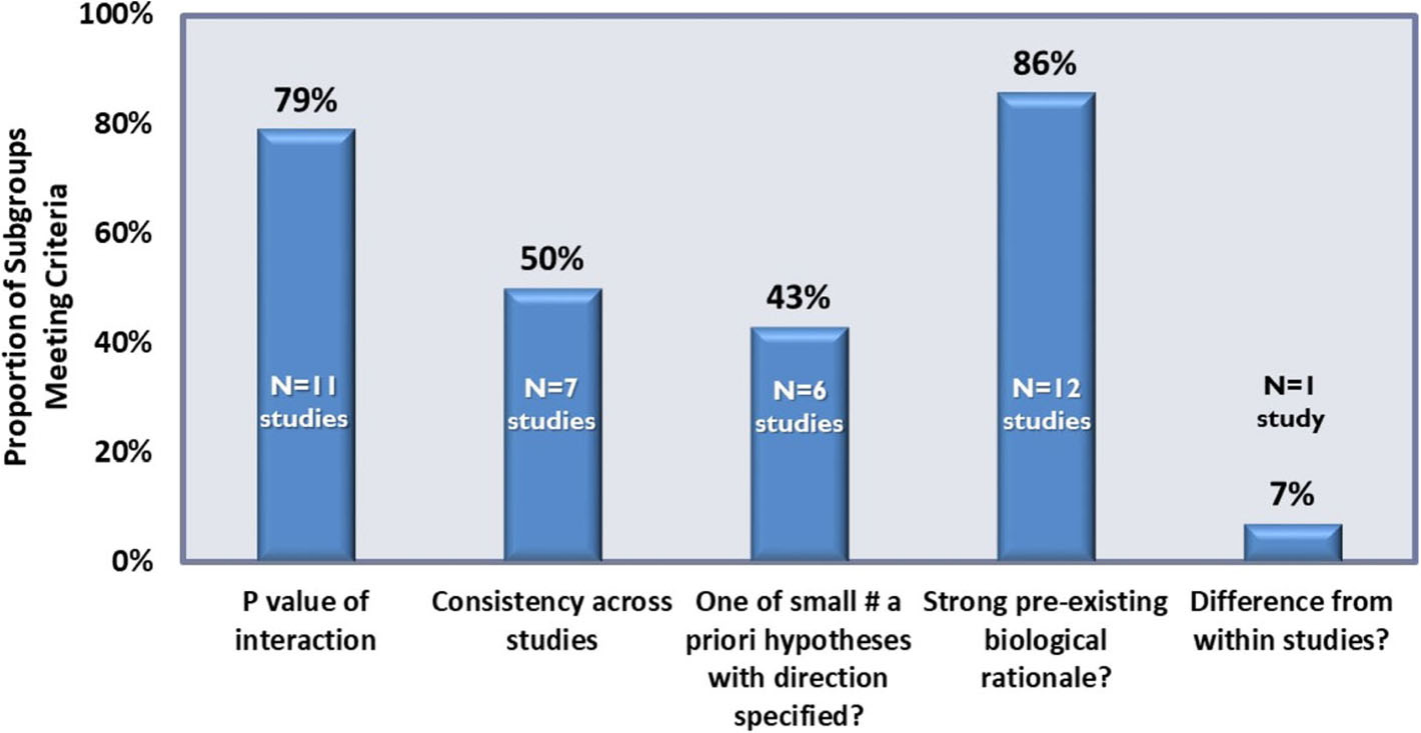
Overall credibility measures of subgroup effects. Credibility is assessed using the criteria established by Sun et. al. [10]

The credibility of subgroup effects was assessed for the 14 subgroup effects identified, with the most credible subgroup effects being stroke risk score by CHADs_2_ [35], sex [39], and age [35] of patients (these met 4 out of 5 subgroup credibility criteria) and many were moderately credible (meeting 3 credibility criteria), including oral anticoagulation type [35], dose [35], and race of patient [35]. The composite ratings for credibility of each of the subgroup effects are presented in Fig. 3 and the proportion of subgroup effects meeting each of the credibility criteria are presented in Fig. 4. Less than half of the subgroup effects met the criteria of being one of a small number of *pre-specified* hypotheses with direction of effect pre-specified (43%), and few subgroup effects (7%) were identified from data within studies. Furthermore, only half (50%) of the subgroup effects were consistent across studies.

Of the 5 reviews that reported subgroup effects, 3 reviews [30, 35, 39] discussed and noted the subgroup effects as part of the overall conclusions and two reviews did not [31, 38]. None of the reviews discussed credibility of subgroup effects in the discussion or conclusions.

## Discussion

This methodological review highlights important gaps in Cochrane systematic reviews of AF. Although numerous subgroup analyses were planned, many could not be conducted due to insufficient studies including important subgroups such as those based on stroke and bleeding risk factors and important co-morbid conditions such as heart failure and diabetes. Similar findings have been reported in Cochrane SRs of HIV, showing that subgroup analyses are often not possible to conduct and subgroup effects are rare [44]. In addition, no reviews included details on how covariates were selected, or if statistical considerations necessitated an abbreviated list of covariates from an initial set pre-specified for investigation. We also found limitations in the quality of the reviews and the credibility of the subgroup effects were not discussed. In an earlier review, it was noted that most Cochrane review authors did not adequately interpret or report subgroup analyses, nor did the review authors discuss the plausibility of effects [12]. Although only observed in a relatively small number of Cochrane reviews, these issues may potentially extend to other reviews in this area and should be further explored (in both primary studies and reviews, including meta-epidemiological reviews).

Guidance for systematic planning of investigations of heterogeneity have been provided, with recommendations to outline these a priori in a protocol, providing a clear rationale with appropriate consideration of the multiple sources and levels of possible heterogeneity, guided by clinical expert opinion [4]. This review has identified gaps in planning and conduct of AF SRs in a subset of Cochrane SRs. Of the subgroup analyses conducted, only half revealed subgroup effects and most of these were not pre-specified which raises the suspicion of selective post hoc reporting. As subgroups identified from a limited number of *pre-specified* hypotheses have greater credibility than those conducted post hoc, this may be an important methodological consideration for future systematic reviews and primary studies. Further important limitations to the subgroup effects identified in these reviews are that most effects were identified between studies rather than within studies and only half of the subgroup effects identified were consistent across studies. There are resources available for SR review authors which can be consulted prior to planning of SRs which may improve interpretation of subgroup analyses [2, 12, 45]. Important topics related to investigation of subgroups in SRs such as issues of confounding, and the inherent observational nature of these analyses should be addressed in the interpretation of any findings [2], issues which may also extend to randomized trials that are included in the reviews [46]. Without these appropriate considerations, the subgroup effects claimed may be misleading.

Although more than half of the reviews were assessed as low quality, there was some evidence that this was driven by our improved understanding and updating of important risk of bias domains assessed in systematic reviews [47] as more recent SRs were more often rated as being of high quality. Of note, the high quality reviews planned more subgroup analyses on average than low quality reviews.

### Subgroup effects identified

This review showed that anticoagulant characteristics including type, route, quality, and dose, although rarely pre-specified for subgroup analysis, may have important differences in treatment effect which should be more broadly evaluated. As head to head comparisons of some treatments such as comparisons within the classes of non-VKA oral anticoagulants (NOAC) are rarely available in clinical trials, systematic reviews could help guide clinical decision-making if credible subgroup analyses show differences in outcomes between anticoagulant types or show that some patient subgroups fare better with one type of NOAC compared to another.

The relatively small number of investigated covariates related to co-morbidities, stroke risk, and AF type suggest that there may be a lack of sufficient data in the primary studies to make these comparisons. It is therefore important to encourage researchers to report data on important subgroups in the primary studies so these can also be more robustly evaluated in systematic reviews.

### Limitations

This meta-epidemiological review of SRs of AF exclusively evaluated Cochrane reviews which represents approximately 15% of SRs conducted [48] and limits the generalizability of findings. Although different subgroups may be identified in journal articles or health technology assessments, the methodological issues identified are likely underestimated if extended to other sources where established protocols for inquiry and reporting are absent. Furthermore, as subgroup analyses are limited to large-scale randomized trials, potentially important data from smaller or single-center studies may have been excluded. Including SRs published in other journals may provide more comprehensive insight into important subgroups which should be examined. The more recent availability of additional NOACs may warrant further evaluation as data becomes increasingly available, compelling publication of additional SRs and potentially a follow-up meta-epidemiological review.

## Conclusion

This meta-epidemiological review of a subset of Cochrane AF reviews suggests that planning and reporting of subgroup analyses in AF reviews can be improved to better inform clinical management. Most pre-specified subgroup analyses in AF SRs were not performed due to insufficient data, and important variables, such as stroke and bleeding risk, and comorbidities were rarely examined. Furthermore, the credibility of subgroups was not assessed or reported in the SRs. These results suggest that more comprehensive planning and reporting of subgroup analyses in Cochrane AF SRs is warranted to ensure the most clinically important subgroups are appropriately identified and interpreted and where possible, important heterogeneity is explained.

Future reviews should aim to identify important subgroups in their protocols and discuss credibility of subgroups effects to better support clinical decision-making.

### Supplementary information

**Additional file 1.** Proposed items to be used for reporting methodology research, adapted from the PRISMA Checklist (http://prismastatement.org/PRISMAStatement/Checklist.aspx)

**Additional file 2.** AMSTAR-2 Risk of Bias for Included Systematic Reviews

## Appendix

Table of systematic reviews

**Table:**

Author (year)	Title	Objectives	Studies included	Trials (*n*)	Patients (*n*)	Primary outcomes	Types of participants	Types of interventions
Huffman et al. [33]	Concomitant atrial fibrillation surgery for people undergoing cardiac surgery	To assess the effects of concomitant AF surgery among people with AF who are undergoing cardiac surgery.	RCTs evaluating the effect of any concomitant AF surgery compared with no AF surgery	22	1899	All-cause mortality, freedom from AF, flutter, or tachycardia. Procedural safety (adverse events)	Adults with pre-operative AF, undergoing cardiac surgery for another indication	Any concomitant AF surgery
Aguilar et al. [26]	Oral anticoagulants versus antiplatelet therapy for preventing stroke in patients with non-valvular AF and no history of stroke or TIA	To characterize relative effect of long-term OAC treatment compared with antiplatelet therapy on major vascular events in patients with non-valvular AF and no history of stroke/TIA.	RCTs in which long-term adjusted-dose OAC was compared with antiplatelet therapy in patients with chronic non-valvular AF.	8	9598	All strokes (ischemic and hemorrhagic)	Non-valvular AF patients, most without previous stroke or TIA	Oral anticoagulation by vitamin K antagonists
Aguilar and Hart [24]	Oral anticoagulants for preventing stroke in patients with non-valvular AF and no previous history of stroke or TIA	To characterize the efficacy and safety of OAC for the primary prevention of stroke in patients with chronic AF.	RCTs comparing OACs with control in patients with chronic non-valvular atrial fibrillation and no history of TIA/stroke.	5	2313	All strokes (ischemic and hemorrhagic)	Non-valvular AF; most without previous stroke or TIA	Oral anticoagulants for preventing stroke in patients with non-valvular AF
Aguilar and Hart [25]	Antiplatelet therapy for preventing stroke in patients with non-valvular atrial fibrillation and no previous history of stroke or TIA	To characterize the effect of long-term antiplatelet on major vascular events in patients with non-valvular AF and no history of stroke or TIA.	RCTs in which long-term antiplatelet was compared to placebo/control in patients with chronic non-valvular AF.	3	1965	All strokes (ischemic and hemorrhagic)	Non-valvular AF without previous stroke or TIA	Aspirin or other specific platelet anti-aggregants given in any dose, formulation, combination, or frequency were considered.
Saxena and Koudstaal [27]	Anticoagulants for preventing stroke in patients with non-rheumatic atrial fibrillation and a history of stroke or TIA	To determine the value of anticoagulant treatment for the long-term prevention of recurrent vascular events in patients with non-rheumatic AF and previous TIA or minor ischaemic stroke.	RCTs - Open-label oral anticoagulants versus control or double-blind anticoagulant treatment versus placebo.	2	485	Composite event of vascular death, nonfatal stroke, nonfatal MI or systematic embolism.	Patients with non-rheumatic AF and a previous history of TIA or minor ischaemic stroke. Only included patients without contraindications to anticoagulants.	Anticoagulation (INR 2.5-4.0 in one study).
Saxena and Koudstaal [28]	Anticoagulants versus antiplatelet therapy for preventing stroke in patients with non-rheumatic atrial fibrillation and a history of stroke or TIA.	To compare the value of anticoagulant and antiplatelet therapy for the long-term prevention of recurrent vascular events in patients with non-rheumatic AF.	RCTs comparing anticoagulants to antiplatelets in patients with prior TIA or stroke.	2	1371	Composite event of vascular death, nonfatal stroke, nonfatal myocardial infarction or systematic embolism.	Patients with non-rheumatic AF and a previous history of TIA or minor ischaemic stroke. Excluded patients with poorly controlled hypertension.	Anticoagulation (INR 2.5-4.0 in one study; 2.4-3.0 in the other).
Kimachi et al. [29]	Direct OAC versus warfarin for preventing stroke and systemic embolic events among atrial fibrillation patients with chronic kidney disease	To assess the efficacy and safety of DOAC including apixaban, dabigatran, edoxaban, and rivaroxaban versus warfarin among AF patients with CKD.	RCTs and quasi-RCTs (RCTs in which treatment allocation was by predictable methods such as date of birth) comparing DOAC with warfarin.	5	12,545	Composite of all strokes and systemic embolic events. Major bleeding or symptomatic bleeding in a critical area or organ.	Eligible participants were diagnosed with non-valvular AF and moderate kidney impairment, defined as CrCl or eGFR between 15 and 60 mL/min.	DTIs including apixaban, dabigatran, edoxaban, and rivaroxaban as well as any other intervention classified as DOAC. Warfarin was to be dose-adjusted using INR.
Lafuente-Lafuente et al. [30]	Antiarrhythmics for maintaining sinus rhythm after cardioversion of atrial fibrillation	To determine in patients who have recovered sinus rhythm after having AF, the effects of long-term treatment with antiarrhythmic drugs on death, stroke, embolism, drug adverse effects and AF recurrence.	RCTs comparing any antiarrhythmic drug with a control in adults who had AF and in whom sinus rhythm was restored.	59	21,305	Mortality, Embolic complications, and adverse events.	Adults (> 16 years) who had AF of any type and duration and in whom sinus rhythm had been restored, spontaneously or by any therapeutic intervention.	Oral long-term treatment with any available antiarrhythmic drug, at an appropriate dosing regime, aimed at preventing new episodes of AF.
Salazar et al. [31]	Direct thrombin inhibitors versus vitamin K antagonists for preventing cerebral or systemic embolism in people with non-valvular atrial fibrillation	To assess comparative safety and efficacy of long-term anticoagulation using DTIs versus VKAs on vascular deaths and ischaemic events in people with non-valvular AF.	RCTs comparing DTIs versus VKAs for prevention of stroke and systemic embolism in people with non-valvular AF.	8	27,557	Vascular death + ischemic events, major bleeding. Composite outcome of vascular deaths and ischaemic events. Composite outcome of fatal or non-fatal major bleeding events.	People with non-valvular AF and one or more risk factors for stroke.	DTIs at standard doses compared with VKAs (adjusted-dose warfarin) for an INR between 2 and 3.
Nyong et al. [32]	Efficacy and safety of ablation for people with non-paroxysmal atrial fibrillation	To determine the efficacy and safety of ablation (catheter and surgical) in people with non-paroxysmal AF compared to antiarrhythmic drugs.	RCTs evaluating the effect of radiofrequency catheter ablation or surgical ablation compared with antiarrhythmic drugs.	3	261	Freedom from atrial arrhythmias, or recurrence of any atrial arrhythmias.	Adult patients with persistent or long-standing persistent AF.	Radiofrequency catheter ablation technique.
Risom et al. [34]	Exercise-based cardiac rehabilitation for adults with atrial fibrillation	To assess benefits and harms of exercise-based rehabilitation programs, alone or with another intervention, compared with no exercise training in adults who currently have AF, or have been treated for AF.	RCT that investigated exercise-based interventions compared with any type of no-exercise control.	6	4 21	Mortality, serious adverse events, health-related quality of life.	Adult patients (18 years old or older) with AF, or who have been treated for AF.	Rehabilitation program with exercise training included. Exercise-based interventions were defined as: any rehabilitation program in an inpatient, outpatient, or community- or homebased setting.
Bruins and Berge [35]	Factor Xa inhibitors versus vitamin K antagonists for preventing cerebral or systemic embolism in patients with AF.	To assess the effectiveness and safety of treatment with factor Xa inhibitors versus VKAs for preventing cerebral or systemic embolic events in people with AF.	RCTs that directly compared effects of long-term treatment with Factor Xa inhibitors vs. VKAs for preventing cerebral and systemic embolism in AF patients.	13	67,688	The composite endpoint of all strokes (both ischaemic and hemorrhagic) and other systemic embolic events.	People with AF who were eligible for treatment with anticoagulants.	Treatment with an oral or parenteral factor Xa inhibitor versus oral vitamin K antagonists (warfarin and congeners) with the intensity monitored using INR.
Moran et al. [39]	Systematic screening for the detection of atrial fibrillation	The primary objective of the review was to investigate whether evidence shows differences between systematic screening and routine practice in the detection of new cases of AF	RCTs and cluster-RCTs comparing systematic screening vs. routine practice.	1	9137	Primary outcome under investigation was the difference in the detection of new cases of AF associated with systematic screening compared with routine practice.	Men and women over the age of 40 years.	This was a diagnostic screening study so no intervention per se. Studies eligible for inclusion compared population-based, targeted or opportunistic screening program versus no screening.
Clarkesmith et al. [36]	Educational and behavioural interventions for anticoagulant therapy in patients with atrial fibrillation	To evaluate the effects of educational and behavioural interventions for OAC on TTR in AF patients.	RCTs evaluating the effect of any educational and behavioral intervention compared with usual care, no intervention, or intervention in combination with other self-management techniques.	11	2246	Primary outcome measure was TTR.	Adults diagnosed with AF	All types of educational and behavioural interventions.
Chen et al. [37]	Catheter ablation for paroxysmal and persistent atrial fibrillation	The primary objective was to assess the beneficial and harmful effects of catheter ablation in comparison with medical treatment in patients with paroxysmal and persistent AF.	RCTs in people with paroxysmal and persistent AF treated by any type of ablation method.	7	3560	Recurrence of AF, fatal and non-fatal embolic complications. All-cause mortality, death due to thrombo-embolic events.	Patients with paroxysmal and persistent AF.	Any type of catheter ablation, including pulmonary vein electrical isolation, superior vena cava isolation, left atrium posterior wall ablation and others.
Mead et al.* [40]	Electrical cardioversion for atrial fibrillation and flutter	To assess effects of cardioversion of AF/ flutter on risk of thromboembolic events, strokes and mortality, the rate of cognitive decline, quality of life, the use of anticoagulants and risk of re-hospitalisation.	RCTs of electrical cardioversion plus ‘usual care’ vs. ‘usual care’ only.	3	927	Primary outcome: risk of stroke, peripheral embolism, and death.	Adults with paroxysmal, sustained or permanent AF or atrial flutter.	Electrical cardioversion used as a first intervention plus ‘usual care’ versus ‘usual care’ only. ‘Usual care’ included drugs for ‘rate control’, anticoagulants or antiplatelet drugs.
Heneghan et al. [38]	Self-monitoring and self-management of oral anticoagulation	To evaluate effects on thrombotic events, major hemorrhages, and all-cause mortality of self-monitoring or self-management of oral anticoagulation compared with standard monitoring.	RCTs assessing the therapeutic effectiveness and safety of self-monitoring or self-management of oral anticoagulation therapy.	28	8950	Primary outcomes were thromboembolic events, all-cause mortality, major haemorrhage, TTR.	All patients on long-term OAC (treatment duration longer than two months), irrespective of the indication for treatment.	Self-monitoring or self-management of oral anticoagulation

*AF* Atrial Fibrillation, *CKD* Chronic Kidney Disease, *CrCl* Creatinine Clearance, *DOAC* Direct Thrombin Inhibitor, *DTI* Direct Thrombin Inhibitor, *eGFR* Estimated Glomerular Filtration Rate, *INR* International Normalized Ratio, *OAC* Oral Anticoagulant, *RCT* Randomized Controlled Trials, *TIA* Transient Ischemic Attack, *TTR* Time in Therapeutic Range

*This review was subsequently withdrawn.

## Abbreviations

AF: Atrial fibrillation
AMSTAR: A MeaSurement Tool to Assess systematic Reviews
CI: Confidence interval
*I*^2^: *I*-squared
IQR: Interquartile range
NOAC: Non-vitamin K anticoagulant
OAC: Oral anticoagulant
OR: Odds ratio
PROSPERO: International Prospective Register of Systematic Reviews
SR: Systematic review
TIA: Transient ischemic attack
VKA: Vitamin K antagonist
