# Supplementary material for: A meta-epidemiological study of subgroup analyses in cochrane systematic reviews of atrial fibrillation
Source: Syst Rev. 2019 Oct 25;8:241. doi: 10.1186/s13643-019-1152-z (PMC6814034; doi:10.1186/s13643-019-1152-z)
Supplement: Supplementary file 1 — Additional file 1. Proposed items to be used for reporting methodology research, adapted from the PRISMA Checklist (http://prismastatement.org/PRISMAStatement/Checklist.aspx) [file 13643_2019_1152_MOESM1_ESM.docx]

**Additional File 1:** Proposed items to be used for reporting methodology research, adapted from the PRISMA Checklist (http://prismastatement.org/PRISMAStatement/Checklist.aspx)^1^

| Section/topic | Proposed item to be used in methodology research | **Manuscript location** |
| --- | --- | --- |
| **Title** | |  |
| Title | Identify the report as a meta-epidemiologic study. | Title: page 1 |
| **Abstract** | |  |
| Structured summary | Provide a structured summary that includes the background of the topic, goal of the study, data sources, method of data selection, appraisal and synthesis methods, results, limitations, conclusions and implications of key findings. | Abstract: page 1-2 |
| **Introduction** | |  |
| Rationale | Describe the rationale for the meta-epidemiological study in the context of what is already known. | Background Subsections “Planning and Interpretation of Subgroup Analyses” and “Subgroup Analyses in Atrial Fibrillation”; pages 5-7 |
| Objectives | Provide an explicit statement of the goal of the meta-epidemiologica l study and the hypothesis being empirically tested. | “Subgroup Analyses in Atrial Fibrillation”; page 7 |
| **Methods** | |  |
| Protocol | Indicate if a protocol exists, if and where it can be accessed (eg, Web address). Registration of a protocol is not mandatory. | Methods section: page 7 |
| Eligibility criteria | Specify study characteristics used as criteria for eligibility with a rationale. | “Eligibility Criteria and Selection”: page 8-9 |
| Information  sources | Describe all information sources (eg, databases with dates of coverage, contact with experts to identify additional studies, Internet searches) and search date. | “Search” section: page 8 |
| Search | Present full electronic search strategy for at least one database, including any limits used, such that it could be repeated. Search is commonly not driven by a clinical question. | “Search” section: page 8 |
| Study selection | Describe the process for selecting studies for inclusion (ie, how many reviewers selected studies, reviewing in duplicate or by single individuals). | “Eligibility Criteria and Selection” and “Data Collection and Analysis”: pages 8-9 |
| Data collection process | Describe method of data extraction from reports (eg, piloted forms, independently, in duplicate) and any processes used for manipulating data or obtaining and confirming data from investigators. | “Data Collection and Analysis”: pages 8-11 |
| Data items | List and define all variables for which data were sought and any assumptions and imputations made. | “Data Collection and Analysis”: pages 9-11 |

| Risk of bias in  individual studies | If risk of bias assessment of individual studies was relevant to the analysis, describe the items used and how this information is to be used during data synthesis. | “Quality of Reviews” section: page 15 |
| --- | --- | --- |
| Summary measures | State the principal summary measures (eg, ratio of risk ratios, difference in means) and explain its meaning and direction to readers. | N/A |
| Synthesis of  results | Describe the statistical or descriptive methods of synthesis including measures of consistency if relevant. If applicable, describe the development of statistical or simulation modelling based on theoretical background. Describe and justify assumptions and computational approximations. Describe methods of additional analyses (eg, sensitivity or subgroup analyses, meta-regression), if done, indicating which were prespecified. | Methods subsection ‘Data Collection and Analysis’: page 9-10 |
| Results | |  |
| Study selection | Give numbers of studies assessed for eligibility and included in the study, with reasons for exclusions at each stage, ideally with a flow diagram. Present a measure of inter-reviewer agreement (eg, kappa statistic). | Results subsection ‘Systematic Review Characteristics’: page 11 and Figure 1 |
| Study  characteristics | For each study, present characteristics for which data were extracted and provide the citations. Clinical characteristics may not always be relevant. | Results subsection ‘Systematic Review Characteristics’: page 11 ‘Subgroup Analyses’: pages 11-13 |
| Risk of bias  within studies | If risk of bias assessment of individual studies was used in the meta-epidemiological analysis, report risk of bias indicators of each study to allow replication of findings. | Results subsection ‘AMSTAR-2 Quality of Reviews’: page 14-15 |
| Results of individual studies | Present data elements used in the meta- epidemiological analysis from each study (results of clinical outcomes may not be relevant). | N/A |
| Synthesis of results | Present results of statistical analysis done, including measures of precision and measures of consistency.  Present validity of assumptions and fit of statistical or simulation modelling, if applicable. | N/A |
| Additional analysis | Give results of additional analyses, if done (eg, sensitivity or subgroup analyses, meta-regression). | Results subsection ‘Credibility of Subgroups’: page 15-16 |
| Discussion | |  |
| Summary of evidence | Summarise the main findings and compare them with existing knowledge about the topic. The quality of evidence may not be relevant; however, investigators should describe their certainty in the results to readers. | Discussion: pages 16-17, paragraphs 1-2 |
| Limitations | Discuss limitations at research methodology level (eg, likelihood of reporting or publication bias). | Limitations section: pages 18-19 |
| Conclusions | Provide general interpretation of the results and implications for future research. Provide any plausible impact on clinical practice. | Conclusion section: page 19 |
| Funding | Describe sources of funding for the methodology research and role of funders. | Declarations: page 21 |
