# Supplementary material for: A meta-epidemiological study of subgroup analyses in cochrane systematic reviews of atrial fibrillation
Source: Syst Rev. 2019 Oct 25;8:241. doi: 10.1186/s13643-019-1152-z (PMC6814034; doi:10.1186/s13643-019-1152-z)
Supplement: Supplementary file 2 — Additional file 2. AMSTAR-2 Risk of Bias for Included Systematic Reviews [file 13643_2019_1152_MOESM2_ESM.docx]

**Additional File 2:** AMSTAR-2 Risk of Bias for Included Systematic Reviews

| First Author Last name | **Huffman**  **2016^1^** | **Aguilar**  **2007^2^** | **Kimachi**  **2017^3^** | **Saxena**  **2004^4^** | **Aguilar** | **Lafuente**  **2015^5^** | **Salazar**  **2014^6^** | **Nyong**  **2016^7^** | **Risom**  **2017^8^** | **Bruins**  **2018^9^** | **Chen**  **2012^10^** | **Moran**  **2016^11^** | **Clarkesmith**  **2017^12^** | **Heneghan**  **2016^13^** | **Aguilar**  **2005^14^** | **Saxena**  **2004^15^** | **Mead**  **2009^16^** |
| --- | --- | --- | --- | --- | --- | --- | --- | --- | --- | --- | --- | --- | --- | --- | --- | --- | --- |
| Year of publication |  |  |  |  | **2005^17^** |  |  |  |  |  |  |  |  |  |  |  |  |
| 1- Did the research questions and inclusion criteria for the review include the components of PICO? | 1 | 1 | 1 | 1 | 1 | 1 | 1 | 1 | 1 | 1 | 1 | 1 | 1 | 1 | 1 | 1 | 1 |
| **2 - Did the report of the review contain an explicit statement that the review methods were established prior to the conduct of the review and did the report justify any significant deviations from the protocol?** | **1** | **1** | **1** | **2** | **2** | **2** | **1** | **1** | **1** | **1** | **1** | **1** | **1** | **1** | **1** | **1** | **1** |
| 3- Did the review authors explain their selection of the study designs for inclusion in the review | 1 | 0 | 0 | 0 | 0 | 0 | 1 | 1 | 1 | 0 | 1 | 0 | 1 | 1 | 1 | 0 | 0 |
| **4- Did the review authors use a comprehensive literature search strategy?** | **2** | **1** | **2** | **1** | **2** | **2** | **2** | **2** | **2** | **1** | **2** | **2** | **1** | **2** | **2** | **1** | **1** |
| 5- Did the review authors perform study selection in duplicate? | 1 | 1 | 1 | 0 | 1 | 1 | 1 | 1 | 1 | 1 | 1 | 1 | 1 | 1 | 1 | 1 | 1 |
| 6 - Did the review authors perform data extraction in duplicate? | 1 | 1 | 1 | 1 | 1 | 1 | 1 | 1 | 1 | 1 | 1 | 1 | 1 | 1 | 1 | 1 | 0 |
| **7 -Did the review authors provide a list of excluded studies and justify the exclusions?** | **1** | **2** | **2** | **0** | **1** | **1** | **1** | **1** | **1** | **1** | **1** | **1** | **1** | **1** | **1** | **0** | **0** |
| 8 -Did the review authors describe the included studies in adequate detail? | 1 | 1 | 1 | 2 | 1 | 2 | 1 | 1 | 1 | 2 | 1 | 1 | 1 | 2 | 1 | 2 | 2 |
| **9 -RCTs: Did the review authors use a satisfactory technique for assessing the risk of bias (RoB) in individual studies that were included in the review?** | **1** | **2** | **1** | **2** | **2** | **2** | **1** | **1** | **1** | **1** | **1** | **1** | **1** | **1** | **2** | **2** | **2** |
| 10 -Did the review authors report on the sources of funding for the studies included in the review? | 1 | 0 | 1 | 0 | 0 | 0 | 1 | 1 | 1 | 1 | 1 | 1 | 1 | 0 | 0 | 0 | 1 |
| **11 -RCTs: If meta-analysis was performed did the review authors use appropriate methods for statistical combination of results?** | **1** | **1** | **1** | **1** | **0** | **1** | **1** | **1** | **1** | **0** | **1** | **1** | **1** | **1** | **1** | **0** | **1** |
| 12 -If meta-analysis was performed, did the review authors assess the potential impact of RoB in individual studies on the results of the meta-analysis or other evidence syntheses? | 1 | 0 | 1 | 0 | 0 | 1 | 0 | 1 | 1 | 1 | 0 | 1 | 0 | 1 | 1 | 0 | 0 |
| **13 -Did the review authors account for RoB in individual studies when interpreting/ discussing the results of the review?** | **0** | **0** | **1** | **1** | **2** | **0** | **1** | **1** | **1** | **1** | **1** | **1** | **1** | **1** | **0** | **1** | **1** |
| 14 -Did the review authors provide a satisfactory explanation for, and discussion of, any heterogeneity observed in the results of the review? | 1 | 1 | 1 | 1 | 1 | 1 | 1 | 1 | 1 | 1 | 1 | 1 | 1 | 0 | 1 | 0 | 0 |
| **15 - If they performed quantitative synthesis did the review authors carry out an adequate investigation of publication bias (small study bias) and discuss its likely impact on the results of the review?** | **1** | **1** | **1** | **1** | **1** | **1** | **1** | **1** | **1** | **1** | **1** | **1** | **1** | **1** | **1** | **1** | **1** |
| 16 - Did the review authors report any potential sources of conflict of interest, including any funding they received for conducting the review? | 1 | 1 | 1 | 1 | 1 | 1 | 1 | 1 | 1 | 1 | 1 | 1 | 1 | 1 | 1 | 1 | 1 |
| **Overall confidence in results of review** | **3** | **3** | **1** | **3** | **3** | **3** | **1** | **1** | **1** | **3** | **1** | **1** | **1** | **1** | **3** | **3** | **3** |

References:

1. Huffman MD, Karmali KN, Berendsen MA, et al. Concomitant atrial fibrillation surgery for people undergoing cardiac surgery. *Cochrane Database of Systematic Reviews* 2016; (8).

2. Aguilar MI, Hart R, Pearce LA. Oral anticoagulants versus antiplatelet therapy for preventing stroke in patients with non‐valvular atrial fibrillation and no history of stroke or transient ischemic attacks. *Cochrane Database of Systematic Reviews* 2007; (3).

3. Kimachi M, Furukawa TA, Kimachi K, Goto Y, Fukuma S, Fukuhara S. Direct oral anticoagulants versus warfarin for preventing stroke and systemic embolic events among atrial fibrillation patients with chronic kidney disease. *Cochrane Database of Systematic Reviews* 2017; (11).

4. Saxena R, Koudstaal PJ. Anticoagulants for preventing stroke in patients with nonrheumatic atrial fibrillation and a history of stroke or transient ischaemic attack. *Cochrane Database of Systematic Reviews* 2004; (2).

5. Lafuente‐Lafuente C, Valembois L, Bergmann JF, Belmin J. Antiarrhythmics for maintaining sinus rhythm after cardioversion of atrial fibrillation. *Cochrane Database of Systematic Reviews* 2015; (3).

6. Salazar CA, del Aguila D, Cordova EG. Direct thrombin inhibitors versus vitamin K antagonists for preventing cerebral or systemic embolism in people with non‐valvular atrial fibrillation. *Cochrane Database of Systematic Reviews* 2014; (3).

7. Nyong J, Amit G, Adler AJ, et al. Efficacy and safety of ablation for people with non‐paroxysmal atrial fibrillation. *Cochrane Database of Systematic Reviews* 2016; (11).

8. Risom SS, Zwisler AD, Johansen PP, et al. Exercise‐based cardiac rehabilitation for adults with atrial fibrillation. *Cochrane Database of Systematic Reviews* 2017; (2).

9. Bruins Slot KMH, Berge E. Factor Xa inhibitors versus vitamin K antagonists for preventing cerebral or systemic embolism in patients with atrial fibrillation. *Cochrane Database of Systematic Reviews* 2018; (3).

10. Chen HS, Wen JM, Wu SN, Liu JP. Catheter ablation for paroxysmal and persistent atrial fibrillation. *Cochrane Database of Systematic Reviews* 2012; (4).

11. Moran PS, Teljeur C, Ryan M, Smith SM. Systematic screening for the detection of atrial fibrillation. *Cochrane Database of Systematic Reviews* 2016; (6).

12. Clarkesmith DE, Pattison HM, Khaing PH, Lane DA. Educational and behavioural interventions for anticoagulant therapy in patients with atrial fibrillation. *Cochrane Database of Systematic Reviews* 2017; (4).

13. Heneghan CJ, Garcia‐Alamino JM, Spencer EA, et al. Self‐monitoring and self‐management of oral anticoagulation. *Cochrane Database of Systematic Reviews* 2016; (7).

14. Aguilar MI, Hart R. Antiplatelet therapy for preventing stroke in patients with non‐valvular atrial fibrillation and no previous history of stroke or transient ischemic attacks. *Cochrane Database of Systematic Reviews* 2005; (4).

15. Saxena R, Koudstaal PJ. Anticoagulants versus antiplatelet therapy for preventing stroke in patients with nonrheumatic atrial fibrillation and a history of stroke or transient ischemic attack. *Cochrane Database of Systematic Reviews* 2004; (4).

16. Mead GE, Elder A, Flapan AD, Cordina J. Electrical cardioversion for atrial fibrillation and flutter. *Cochrane Database of Systematic Reviews* 2017; (11).

17. Aguilar MI, Hart R. Oral anticoagulants for preventing stroke in patients with non‐valvular atrial fibrillation and no previous history of stroke or transient ischemic attacks. *Cochrane Database of Systematic Reviews* 2005; (3).
